# Sinomenine Hydrochloride Attenuates Renal Fibrosis by Inhibiting Excessive Autophagy Induced by Adriamycin: An Experimental Study

**DOI:** 10.1155/2017/6878795

**Published:** 2017-07-17

**Authors:** Ming-ming Zhao, Bin Yang, Qiu Zhang, Jin-hu Wang, Jin-ning Zhao, Yu Zhang

**Affiliations:** Xiyuan Hospital, China Academy of Chinese Medical Sciences, Beijing 100091, China

## Abstract

The objective of this study is to investigate if sinomenine hydrochloride (SIN-HCl) could be effective against adriamycin-induced renal fibrosis by regulating autophagy in a rat model. Forty male Sprague-Dawley (SD) rats were randomly divided into control group, model group, telmisartan group, and SIN-HCl group; rat model was induced by adriamycin; all rats were given intragastric administration for 6 weeks. Urine was collected from rats in metabolic cages to determine 24 h protein level. This was done after intragastric administration for the first two weeks and then once for every two weeks. Renal pathological changes were examined by the staining of HE, Masson, and PASM. Expressions and distributions of fibronectin (FN), laminin (LN), light chain 3 (LC3), and Beclin-1 were observed by immunohistochemistry. SIN-HCl ameliorates proteinuria, meanwhile attenuating the renal pathological changes in adriamycin-induced rats and also attenuating renal fibrosis and excessive autophagy by reducing the expression of FN, LN, LC3, and Beclin-1. SIN-HCl attenuates renal fibrosis by inhibiting excessive autophagy induced by adriamycin and upregulates the basal autophagy.

## 1. Introduction

**Figure 1:**
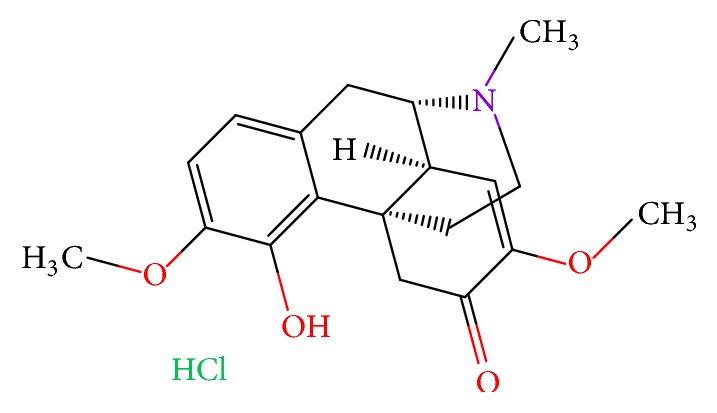
The structure of sinomenine hydrochloride (SIN-HCl).

Chronic kidney disease (CKD) is characterized by reduced glomerular filtration rate, increased urinary albumin excretion, or both [1]. With the increase in the incidence of hypertension, diabetes, and obesity, the number of patients with CKD and end-stage renal disease (ESRD) is expected to rise in the future. Renal fibrosis is regarded as the common pathological alteration in virtually every progressive CKD [2]. With kidney injury, physiological wound healing response appears to restore normal function and tissue homeostasis. However, dysregulation of this response leads to excessive, pathological deposition of extracellular matrix (ECM) proteins such as fibrillar collagens, FN, and LN. The research of renal autophagy is still in the initial stage compared with disciplines such as oncology and neurology [3]. Autophagy in renal diseases can be either protective or detrimental depending on experimental conditions. During early disease stages, maintaining podocyte autophagic activity is a potential therapeutic strategy in the treatment of delaying the progression of podocytopathies [4]. On the contrary, excessive autophagy has been proposed to mediate autophagic cell death [5]. There is a relevance between renal fibrosis and autophagy proven by recent evidences [6]. Sinomenine (Figure 1) is an active alkaloid extracted from the roots of the Chinese medicinal plant* Sinomenium acutum*, which has been employed to treat rheumatoid arthritis and other inflammatory disorders. Pharmacological profiles of sinomenine include immune-modulating properties [7, 8], anti-inflammation [9, 10], and anti-oxidative [11, 12] stress; SIN also contains renoprotective feature.

Based on these discoveries, we hypothesized that SIN-HCl could have similar mechanism to sinomenine; meanwhile, SIN-HCl could be effective against adriamycin-induced renal fibrosis by regulating autophagy in a rat model.

## 2. Materials and Methods

### 2.1. Animals Grouping and Treatment

Forty male SPF Sprague-Dawley (SD) rats (220 ± 18 g) were purchased from Beijing HFK Bioscience Co., Ltd., Beijing, China. Rats were housed in humidity controlled (60 ± 10)% rooms at (24 ± 1)°C with a 12 h on/12 h off light cycle. The animals were maintained with free access to standard diet and tap water.

All rats were habituated to laboratory conditions for 7 days. Forty SD rats were randomly divided into either control group (*n* = 10) or adriamycin-induced group (*n* = 30). Adriamycin-induced group was treated with adriamycin at a dose of 6.2 mg/kg [13] by tail vein injection once, whereas the control group was treated with normal saline at the same dose as adriamycin-induced group by tail vein injection once. The adriamycin-induced group was further assigned to model group (*n* = 10), telmisartan group (*n* = 10), and SIN-HCl group (*n* = 10). Two weeks after being treated with adriamycin, 4 groups were given intragastric administration for 6 weeks: rats in control group and model group were given normal saline, rats in telmisartan group were given telmisartan (8.33 mg/kg), and rats in SIN-HCl group received SIN-HCl (18.75 mg/kg). All drugs were diluted with distilled water, and the dosages were evaluated with body surface coefficient conversion between human and rat. Urine was collected from rats in metabolic cages to determine 24 h protein level. It was done after intragastric administration for the first two weeks and then once for every two weeks. Rats were then sacrificed for the harvesting of kidney tissues. The Animal Care and Use Committee of Xiyuan Hospital of China Academy of Chinese Medical Sciences approved the experimental protocol.

### 2.2. Drugs

SIN-HCl (Zhengqing Fengtongning Tablet, 20 mg/pil, SFDA Approval number Z10980043) was purchased from Zhengqing Pharmaceutical Group (Hunan, China). Telmisartan (Micardis, 80 mg/pill) was purchased from Boehringer Ingelheim International GmbH Z10980043 (Import Drug License: H20090416, H20090417). Doxorubicin Hydrochloride for Injection (instant) was purchased from Pfizer Italia s.r.l. (Import Drug License: H20080278; imported drug registration standards: Jx20000016). Immunohistochemistry antibody was purchased from Abcam Companies (Item number ab52167 and ab32351); secondary antibody (PV-6000) is a general-purpose two-step immunohistochemical kit; DAB kit was purchased from ZSGB Biological Technology Co., Ltd.

### 2.3. Histopathological Analysis

Renal cortex tissues of right kidney were fixed in 10% buffered formalin, then embedded in paraffin, and sliced. Slices were stained with haematoxylin-eosin (HE), Masson, and methenamine silver stain (PASM), respectively, and visualized using light microscopy. The cortex tissues of left kidney were stored at −70°C for further study.

### 2.4. Immunohistochemistry

Expressions and distributions of FN, LN, LC3, and Beclin-1 were observed by immunohistochemistry. Renal cortical slices were dewaxed and rehydrated. Slices were incubated with 3% H_2_O_2_ and washed with PBS. Then slices were incubated with primary antibodies at 4°C for overnight and washed with PBS. The slices were incubated with secondary antibodies and washed with PBS. The slices were stained with DAB, washed, dehydrated, permeabilized, mounted, and viewed by light microscopy. The expression of protein was demonstrated by the ratio of integral optical density (IOD): IOD = average optical density × positive area.

## 3. Statistical Analysis

Statistical analysis was performed by one-way ANOVA analysis of variance using SPSS software 20.0 (SPSS, Chicago, USA). The data are expressed as mean ± standard deviation (SD). *P* < 0.05 was considered a statistically significant difference, and all *P* values were two-sided.

## 4. Results

### 4.1. SIN-HCl Ameliorates Proteinuria

**Figure 2:**
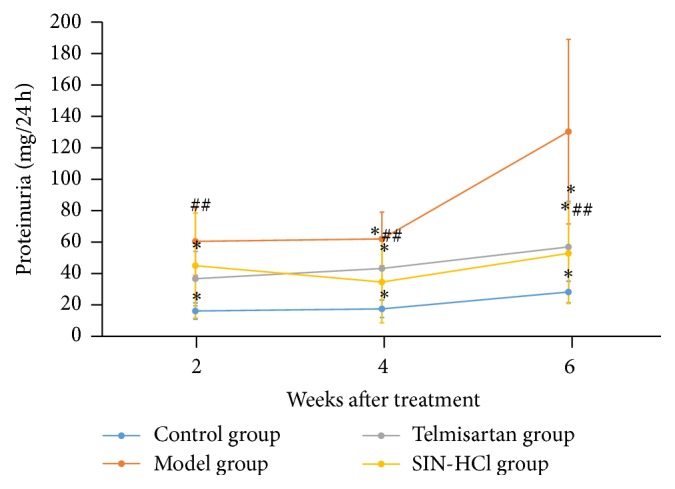
SIN-HCl ameliorated proteinuria. Data are shown as mean ± SD; ^*∗*^*P* < 0.05 versus model group; ^##^*P* > 0.05 versus telmisartan group. At the second week, 24 h proteinuria significantly decreased after being treated with telmisartan. At the fourth and sixth week, 24 h proteinuria significantly decreased in the telmisartan group and SIN-HCl group; meanwhile, there was no difference between telmisartan group and SIN-HCl group.

As shown in Figure 2, rats injected with adriamycin developed proteinuria compared with control group; proteinuria continually increased. At the second week of being treated with SIN-HCl, there was no difference in 24 h proteinuria between model group and SIN-HCl group. However, 24 h proteinuria significantly decreased after four-week SIN-HCl treatment. At the fourth and sixth week, 24 h proteinuria significantly decreased in the telmisartan group and SIN-HCl group; meanwhile, there was no difference between telmisartan group and SIN-HCl group.

### 4.2. SIN-HCl Attenuates Renal Pathological Changes

**Figure 3:**
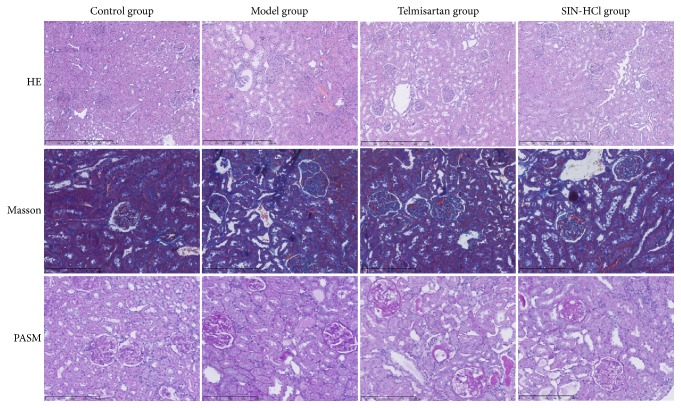
Telmisartan and SIN-HCl ameliorated the renal pathological changes in adriamycin-induced rats. Proliferative mesangial cells, increased ECM, thickened glomerular basement membrane, and disorderly arranged renal tubular cells were showed in the model group. Compared with model group, telmisartan and SIN-HCl ameliorated the renal pathological changes in adriamycin-induced rats.

As shown in Figure 3, renal pathological changes were examined by the staining of HE, Masson, and PASM. Proliferative mesangial cells, increased ECM, thickened glomerular basement membrane, and disorderly arranged renal tubular cells were shown in the model group. Compared with model group, telmisartan and SIN-HCl ameliorated the renal pathological changes in adriamycin-induced rats. The result proved the protective effect of SIN-HCl on kidney in adriamycin-induced rats.

### 4.3. SIN-HCl Attenuates Renal Fibrosis

**Figure 4:**
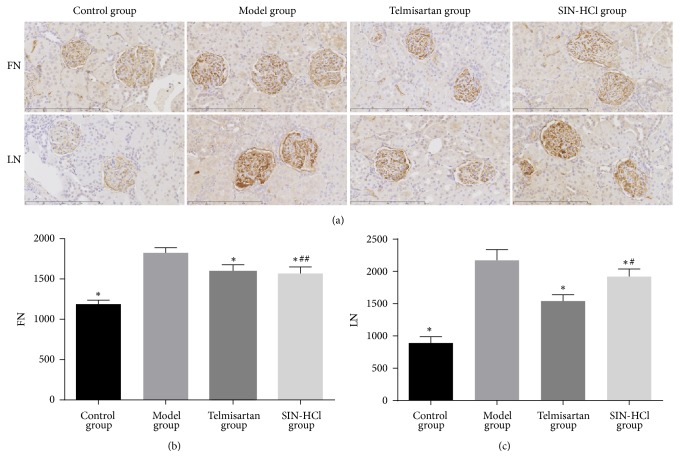
Telmisartan and SIN-HCl attenuated renal fibrosis (FN, LN ×400). FN: fibronectin; LN: laminin. (a) The expression of FN and LN in adriamycin-induced rat glomeruli by immunohistochemistry. (b, c) Quantitation of FN and LN. Data are shown as mean ± SD, ^*∗*^*P* < 0.05 versus model group; ^#^*P* < 0.05 versus telmisartan group; ^##^*P* > 0.05 versus telmisartan group.

To test the effect of SIN-HCl on renal fibrosis, we examined the expression of FN and LN protein levels, as shown in Figure 4. As a result of immunohistochemistry, the expression levels of FN (Figure 4(b)) and LN (Figure 4(c)) in glomeruli were significantly decreased in adriamycin-induced rats treated with SIN-HCl or telmisartan. There was no statistically significant difference in the expression levels of FN between SIN-HCl group and telmisartan group, but telmisartan inhibited the expression of LN superior to SIN-HCl. These results showed that SIN-HCl ameliorated the renal fibrosis in adriamycin-induced rats.

### 4.4. SIN-HCl Attenuates Excessive Autophagy

**Figure 5:**
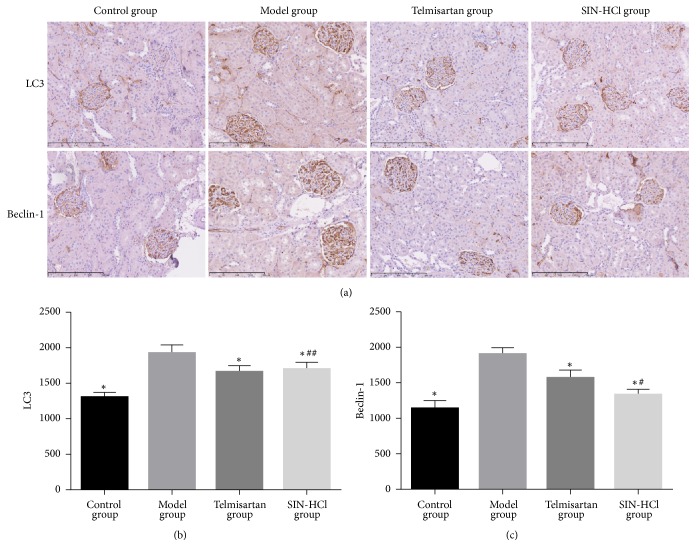
Telmisartan and SIN-HCl attenuated excessive autophagy (LC3, Beclin-1 ×400). (a) The expression of LC3 and Beclin-1 in adriamycin-induced rat glomeruli by immunohistochemistry. (b, c) Quantitation of LC3 and Beclin-1. Data are shown as mean ± SD, ^*∗*^*P* < 0.05 versus model group; ^#^*P* < 0.05 versus telmisartan group; ^##^*P* > 0.05 versus telmisartan group.

To test the effect of SIN-HCl on excessive autophagy, we examined the expression of LC3 and Beclin-1 by immunohistochemistry (Figure 5). The results demonstrated that adriamycin induced increased LC3 (Figure 5(b)) and Beclin-1 (Figure 5(c)), whereas the increment was inhibited after being treated with SIN-HCl or telmisartan. The difference between control group and SIN-HCl group on the expression of Beclin-1 was not statistically significant. These results showed that SIN-HCl ameliorated the excessive autophagy in adriamycin induced rats and upregulated the basal autophagy.

## 5. Discussion

Adriamycin-induced nephropathy is one of rat models of CKD which is considered to induce a nephrotic syndrome, leading to chronic proteinuric renal diseases and renal fibrosis [14, 15]. Clinical and laboratory-based studies have suggested that telmisartan reduces proteinuria in CKD patients [15] and attenuates oxidative stress and renal fibrosis [16, 17]. In this study, the result demonstrated the protective effective of SIN-HCl on adriamycin-induced renal injury. After being treated with adriamycin, rats developed proteinuria and the renal pathological changes were aggravated, such as proliferative mesangial cells, increased ECM, thickened glomerular basement membrane, and disorderly arranged renal tubular cells. SIN-HCl ameliorates proteinuria and renal pathological changes induced by adriamycin.

Renal fibrosis [18] is a pathological process and the common end point of virtually all kidney diseases which should be viewed as a dynamic system that involves mesangial cell, ECM, and many other cells. Glomerular sclerosis and interstitial fibrosis are characteristic lesions in different forms of progressive renal diseases; these pathological lesions are caused by the accumulation of ECM mainly. The ECM is composed of FN and LN mainly, and FN provides support for deposition of ECM components and formation of collagen. Meanwhile, interstitial fibroblast activation and subsequent differentiation into ECM-producing myofibroblasts also are an approach to aggravation of renal fibrosis [19–21]. For illustrating the effect of SIN-HCl on renal fibrosis, we examined the expression of FN and LN in the renal cortical of adriamycin-induced rats. The result demonstrated that SIN-HCl attenuates renal fibrosis by reducing the expression of FN and LN.

Autophagy is the degradation system and dynamic recycling system in cells, cytoplasmic materials are transported and delivered to lysosomes for degradation [22] and, subsequently, new building components and energy produced for cellular renovation and homeostasis. Autophagy includes microautophagy, macroautophagy, and molecular chaperone-mediated autophagy; macroautophagy is considered as the major type of autophagy. LC3 and Beclin-1 are markers of autophagy; LC3 is the essential macroautophagy protein [23]. Aberrant autophagy could cause renal injury and aggravation of various renal diseases. In this study, SIN-HCl attenuates excessive autophagy in kidney by downregulating the excessive expression of LC3 and Beclin-1, meanwhile upregulating the basal autophagy.

Accumulating evidence showed a link between renal fibrosis and autophagy, but the role of autophagy in CKD is complex; induction of autophagy may be a cytoprotective mechanism, thereby negatively regulating ECM, but autophagy also can promote renal fibrosis depending on experimental conditions and cellular context [6]. The persistent and active autophagy of renal tubules aggravated renal interstitial fibrosis. The profibrotic function of autophagy is based on the regulation on tubular cells death, interstitial inflammation, and the production of profibrotic factors [24], whereas the lack of autophagy-related factors could cause the accumulation of FN and the development of renal fibrosis [25]. We found that SIN-HCl not only attenuates renal fibrosis but also attenuates excessive autophagy in renal injury caused by adriamycin; basal autophagy was also upregulated by SIN-HCl. We postulated that SIN-HCl attenuates renal fibrosis by inhibiting excessive autophagy induced by adriamycin and upregulates the basal autophagy.

Pharmacological profiles of sinomenine in treatment of CKD are of multitarget.Sinomenine could activate Nrf2 signaling to prevent hyperactive inflammation and kidney injury [26], meanwhile attenuating renal fibrosis through Nrf2-mediated inhibition of oxidative stress and TGF-*β* signaling [11] and alleviating high glucose-induced renal glomerular endothelial hyperpermeability [27]. However, there were some limitations in the study. Firstly, we could not identify the definite relationship between renal fibrosis and autophagy, due to the lack of autophagy inhibitor group in rats. Secondly, basal autophagy is different in different renal cells, such that the basal autophagy of podocyte is high; however, tubular cells display lower basal autophagy; SIN-HCl regulates autophagy should be verified in vitro.

## 6. Conclusion

In conclusion, SIN-HCl ameliorates proteinuria and, meanwhile, attenuates renal fibrosis by inhibiting excessive autophagy in adriamycin-induced nephropathy and upregulating the basal autophagy. For a future study, based on improvement of grouping, we plan to explore the relation between renal fibrosis and autophagy with SIN-HCl in vivo and in vitro.

## Abbreviations

SIN-HCl: Sinomenine hydrochloride
SD: Sprague-Dawley
FN: Fibronectin
LN: Laminin
LC3: Light chain 3
CKD: Chronic kidney disease
ESRD: End-stage renal disease
ECM: Extracellular matrix
HE: Haematoxylin-eosin
PASM: Methenamine silver stain
IOD: Integral optical density
SD: Standard deviation.
